# Lived Experience of Extracorporeal Membrane Oxygenation Survivors: A Phenomenological Study

**DOI:** 10.1111/nicc.70341

**Published:** 2026-02-01

**Authors:** Meng Zhang, Anqi Sun, Anqi Yu, Juan Deng, Yi Wang, Xiao Cheng, Jie Xiong

**Affiliations:** ^1^ Department of Rehabilitation Medicine Wuhan Hospital of Traditional Chinese and Western Medicine Wuhan Hubei China; ^2^ School of Nursing, Tongji Medical College Huazhong University of Science and Technology Wuhan Hubei China; ^3^ Department of Nursing The Second Affiliated Hospital, Zhejiang University of Medicine Hangzhou Zhejiang China; ^4^ Department of Intensive Care Unit Tongji Hospital, Tongji Medical College of Huazhong University of Science and Technology Wuhan Hubei China

**Keywords:** extracorporeal membrane oxygenation, health‐related quality of life, HRQoL, phenomenological study, qualitative study, survival experience

## Abstract

**Background:**

Extracorporeal membrane oxygenation (ECMO) is a vital extracorporeal life support for acute cardiopulmonary failure, serving as a critical lifesaving measure widely utilised during the COVID‐19 pandemic. Despite the enhancement of survival rates and the extension of its utilisation, ECMO survivors face complications and post‐discharge challenges. Understanding their lived experience is crucial to promote nursing quality improvement and improve their health‐related quality of life (HRQoL).

**Aim:**

To explore the lived experiences of ECMO survivors.

**Study Design:**

A phenomenological study was conducted among patients who had survived ECMO treatment at Tongji Hospital in Wuhan between 2013 and 2023. Interview guides and data analysis were informed by the biopsychosocial medical model. The interview data were analysed using the Giorgi data analysis method.

**Findings:**

In total, 16 ECMO survivors participated. Three themes and 12 sub‐themes emerged: (1) Living conditions and challenges (physical limitations, long‐term effects of complications, lifestyle adjustments, self‐rehabilitation and exercise, family economic burden); (2) Emotional distress and support (emotional difficulties and psychological challenges, emotion management and support perception); (3) Expectations and needs (desire for expanded insurance reimbursement, eagerness for rehabilitation knowledge, hope for simplified follow‐up procedures and normalisation of regular follow‐up).

**Conclusions:**

ECMO survivors face multiple challenges and needs during their recovery. To improve their HRQoL and well‐being, healthcare providers should closely monitor the psychological status, pay attention to their complaints and feelings, and provide targeted nursing guidance and multidimensional social support.

**Relevance to Clinical Practice:**

The lived experience of ECMO survivors is complex and their rehabilitation is a long process requiring comprehensive support to assist them in better integrating into society.

## Impact Statements

- What is known about the subject
  ○ ECMO is a vital life support technology increasingly used in clinical practice, significantly improving survival rates among patients with severe cardiopulmonary failure.
  ○ ECMO survivors often experience long‐term physical, emotional and cognitive challenges that impair their HRQoL.
  ○ Existing research has predominantly focused on clinical outcomes and mortality predictors, with limited qualitative insight into patients' lived experiences post‐ECMO.
- What the paper adds
  ○ This study provides an in‐depth, multidimensional exploration of the lived experiences of ECMO survivors.
  ○ It identifies key themes such as emotional adaptation, persistent physical limitations and the needs for structured post‐discharge support.
  ○ The findings offer evidence‐based implications for developing tailored rehabilitation programs and continuous care models to improve long‐term recovery and HRQoL for ECMO survivors.

## 1 Introduction

Extracorporeal Membrane Oxygenation (ECMO) is an advanced and continuous extracorporeal life support technology, serving as a core therapeutic intervention for severe cardiopulmonary failure. It is widely used in managing acute and critical conditions, including cardiogenic shock, respiratory failure, cardiopulmonary transplantation and extracorporeal cardiopulmonary resuscitation [1]. ECMO gained broader recognition during the COVID‐19 pandemic in 2019, when it was incorporated into international treatment guidelines and extensively applied in clinical practice [2]. With continuous technological advancements and accumulated clinical experience, the use of ECMO has expanded, leading to improved survival rates among patients [3, 4, 5]. Consequently, clinical outcomes and risk factors associated with mortality in ECMO patients have been increasingly documented [6, 7, 8, 9]. Survivors often face a range of complications such as haemorrhage, stroke and leg injuries and may experience long‐term challenges including emotional disturbances, memory deficits, chronic pain and limitations in daily activities due to the invasive nature of ECMO therapy [1, 6, 7]. These issues contribute to varying degrees of impairment in health‐related quality of life (HRQoL), which encompasses an individual's perceived physical, mental, social and functional well‐being in the context of their health status [10].

While several studies have focused on survival outcomes, nursing demands and predictors of adverse prognosis in ECMO patients [11, 12, 13, 14, 15], there remains limited understanding of the lived experiences of ECMO survivors from a holistic perspective [16]. Therefore, grounded in the biopsychosocial model of health, this study employs semi‐structured interviews to explore the lived experiences of ECMO survivors [17], with the aim of informing the development and implementation of continuous care strategies and scientifically grounded nursing management plans for this population.

## 2 Methods

### 2.1 Study Design

In this study, the descriptive phenomenological method [18] was adopted to conduct semi‐structured interviews with adult ECMO survivors. Based on the study team's experience and existing literature [16, 17, 18, 19], the interview guide (see Supporting Information, Appendix S1) was developed using the ‘bio‐psycho‐social’ medical model [17], and an open‐ended question, ‘Is there anything else you would like to add or share about your experience?’, was included to allow for additional information. The research reports adhere to the Comprehensive Standard for Qualitative Research Reports (COREQ) to ensure transparency [20].

### 2.2 Sample and Recruitment

Purposive sampling was employed in the semi‐structured interviews to maximise diversity. Participants were included in the study if they: (1) underwent ECMO therapy at Tongji Hospital; (2) were discharged for more than 1 month; and (3) provided informed consent and voluntary participation in this study. Participants were excluded from the study for the following reasons: (1) cognitive impairment and psychiatric disorders; and (2) terminal illness or irreversible conditions despite intensive treatment.

Eligible participants were contacted by phone or in person. Interviews were scheduled at participants' convenience. Sampling, data collection and analysis occurred concurrently until data saturation was reached, confirmed by four additional interviews yielding no new themes [21]. All the contacted participants participated in the interviews.

### 2.3 Data Collection

In this study, a semi‐structured qualitative interview was conducted face‐to‐face. All interviews were carried out by two researchers, who had received training in qualitative methods. The first author (MZ) led the interviews to ensure consistency. The other author (AS) was responsible for note‐taking and observation. Interviews were scheduled at participants' convenience to avoid interruptions and were held in quiet, comfortable, familiar settings. During the interviews, the researcher primarily listened and adjusted questions based on their responses. Evaluative or leading questions were avoided, and any ambiguous statements were clarified with participants to ensure accuracy. Interviews were conducted in Mandarin and lasted approximately 30–40 min. With the participants' permission, all interviews were audio‐recorded, and the recordings were transcribed within 24 h after the interview and saved as text. The co‐author (AY) returned the transcribed text to the participants for verification and confirmation.

### 2.4 Data Analysis

The data analysis was conducted concurrently with data collection. Giorgi's descriptive phenomenological method was used to organise and analyse the qualitative data through NVivo 11.0 [22, 23]. The process involved: (1) repeatedly reading transcripts for a holistic understanding; (2) identifying and segmenting meaning units; (3) clustering similar units to extract themes; and (4) synthesising themes into a structural description of the lived experience. The audio recordings were transcribed verbatim and then translated into English by two bilingual researchers. To ensure conceptual accuracy and cultural nuance, a back‐translation procedure was performed, and any discrepancies were resolved through discussion within the research team.

### 2.5 Rigour

Throughout the research process, the trustworthiness of findings was ensured by adhering to Lincoln and Guba's framework, encompassing credibility, dependability, confirmability and transferability [24]. To build credibility, a trust relationship with participants was established before interviews. Furthermore, active listening and posing open‐ended questions were utilised to capture the depth and detail of participants' experiences to enhance credibility. Purposive sampling with maximum variation was employed and context was thickly described to support transferability. To ensure dependability, we documented all procedures, held group discussions and expert reviews. Furthermore, the qualitative research report checklist was used for final verification [20]. Confirmability was achieved through maintaining reflexivity journals, keeping analytic memos, triangulating data across sources and researchers and preserving a full audit trail [25].

### 2.6 Ethics Statement

This study was conducted in accordance with the ethical guidelines of the Declaration of Helsinki and approved by the Hospital Ethics Committee (TJ‐IRB20230730) (Approval Date: 4 July 2023). Participation was voluntary, with the right to withdraw at any time. To ensure confidentiality, all data were anonymised by replacing names with identification codes and were used strictly for research purposes.

## 3 Findings

Sixteen ECMO survivors were interviewed. Participant demographics are detailed in Table 1. Three themes and 12 sub‐themes were identified: (1) living conditions and challenges (physical limitations, long‐term effects of complications, lifestyle adjustments, self‐rehabilitation and exercise, family economic burden); (2) emotional distress and support (emotional difficulties and psychological challenges, emotion management and support perception); (3) expectations and needs (desire for expanded insurance reimbursement, eagerness for rehabilitation knowledge, hope for simplified follow‐up procedures and normalisation of regular follow‐up; see Table 2).

**TABLE 1:** Demographic characteristics of participants.

Characteristic	Mean (range) or *n* (%)
Age, years	40 (15–64)
Gender	
Male	10 (62.50%)
Female	6 (37.50%)
Education level	
No Schooling	1 (6.25%)
Secondary vocational	1 (6.25%)
Junior high school	6 (37.50%)
Higher vocational	1 (6.25%)
High school	4 (25.00%)
Undergraduate	3 (18.75%)
Chronic diseases	
Yes	5 (31.25%)
No	11 (68.75%)
At work/school	
Yes	6 (37.50%)
No	10 (62.50%)
ECMO model	
VV‐ECMO	6 (37.50%)
VA‐ECMO	10 (62.50%)
Length of discharge, month	39.69 (2–120)

**TABLE 2:** Overview of the study themes and sub‐themes and supporting evidence.

Themes	Sub‐themes	Illustrative quotes
Living conditions and challenges	Physical limitations	‘After ECMO treatment, I feel my breathing is not as good as before. I get tired more easily and often have no energy, just a general feeling of weakness. I still go to school as usual, but I can't take P.E. class. It's fine if I'm just sitting quietly.’ (P15)
	Long‐term effects of complications	‘I still can't lift my leg up to now. To put on shoes and socks, I have to lift my leg by hand. Otherwise, I won't even be able to even put on shoes and socks.’ (P9)
	Lifestyle adjustment	‘I will pay attention to my diet by ensuring it provides the necessary nutrition for my body. I also plan to learn about traditional Chinese medicine to better support my recovery. In particular, I will avoid foods with strong or heavy flavors that may hinder the recovery.’ (P11)
	Self‐rehabilitation and exercise	‘I feel like I'm getting better, so I ask my wife to hold me up and take me for a slow walk. Afterward, I notice that I recover more quickly.’ (P4)
	Family economic burden	‘I was in the ICU for 28 days, which cost a lot of money, a huge expense for our rural family. Now our family's economic situation is depressed. My son couldn't support the household on his own, so I started looking for work to help ease the burden.’ (P4)
Emotional distress and support	Emotional difficulties and psychological challenges	‘There will be mood fluctuations and low periods. Most of the time, life feels uninteresting because I'm unable to feel happiness, and perhaps sometimes I am numb.’ (P15)
	Emotion management	‘Engaging in physical activities refreshes me and helps keep my mood positive. Even though I don't have a job and spend most of my time at home with a little pressure, I still maintain a good state of mind.’ (P8)
	Support perception	‘The village has established a poor household assistance program for us, providing certain economic subsidies. I am very grateful to the government.’ (P13)
Expectations and needs	Desire for expanded insurance reimbursement	‘From what I understand, the medical insurance reimbursement is limited. It would be helpful if there could be an additional subsidy on top of the reimbursement rate.’ (P1)
	Eagerness for rehabilitation knowledge	‘In fact, I would particularly like to know what precautions I should take after being discharged from the hospital. I also strongly hope that the hospital could provide more educational materials about disease management. While I can search for information online, the content available on the Internet often lacks authority and reliability.’ (P3)
	Hope for simplified follow‐up procedures	‘The review process can sometimes take an entire day, imposing a considerable burden on patients, particularly those who have traveled from other provinces. It should not be overly complicated.’ (P11)
	Normalisation of regular follow‐up	‘It has been many years since I went on ECMO, and I'm so happy you're here for the follow‐up! Please keep in contact with me usually. I can ask you any questions and that will really help with my recovery.’ (P6)

### 3.1 Theme 1: Living Conditions and Challenges

#### 3.1.1 Physical Limitations

Although the survivors have been discharged from the hospital for a long time and can take care of themselves, their energy and physical strength have remained diminished compared to pre‐ECMO levels, with no significant improvement.

*I couldn't walk when I came back from the hospital. Now I can do anything, but my strength is not as good as before*. *(P4)*

*I can manage daily activities and do housework, but I can't do heavy work*. *(P6)*

*After ECMO treatment, I feel my breathing is not as good as before. I get tired more easily and often have no energy, just a general feeling of weakness. I still go to school as usual, but I can't take P.E. class. It's fine if I'm just sitting quietly*. *(P15)*

#### 3.1.2 Long‐Term Effects of Complications

ECMO survivors face a spectrum of long‐term physical complications, from localised discomfort to profound neurological impairment. These persistent sequelae significantly impair functional capacity and HRQoL, imposing a substantial health burden on them.

Some survivors reported ongoing complications at cannulation sites, characterised by persistent somatic symptoms that impacted daily comfort and function.

*I still can't lift my leg up to now. To put on shoes and socks, I have to lift my leg by hand. Otherwise, I won't even be able to even put on shoes and socks*. *(P9)*

*Now I always have to take anticoagulants and a variety of drugs, which leads to some problems in the body, including heart problems. I feel like a walking medicine cabinet, and I have to be very careful about everything*. *(P10)*

*My scar doesn't need to look perfect, I just want the pain and itching to stop after treatment. Over these months, it has kept growing, becoming a raised, painful, and itchy lump… It affects my sleep. I have to lie on my side to relieve the chest discomfort so I can fall asleep*. *(P14)*

For some survivors, the severity of neurological damage rendered them unable to walk independently, necessitating reliance on wheelchairs, marking the most profound level of functional impact.

*I'm in a wheelchair all the time because I have no strength in my right leg*. *(P9)*

*I had no feeling or strength in my left leg, so when I walked, all the pressure was on my right leg. If I walked for a long time, both legs would hurt. That's why I'm in a wheelchair most of the time—it's a lot easier*. *(P13)*

#### 3.1.3 Lifestyle Adjustments

Participants said that although they could manage self‐care after a recovery period, their lifestyles underwent significant changes. A marked decline in energy and physical strength precluded engagement in high‐intensity activities or heavy labour, necessitating considerable lifestyle adjustments. Furthermore, some participants reported paying greater attention to dietary habits and adopting healthier lifestyles following their illness.

*I currently work in real estate. I didn't particularly enjoy it, but I had to take the job since it was the only option that required minimal physical activity*. *(P5)*

*Now I don't have a job. I just stay at home*. *(P6)*

*I always monitor whether my high blood fat is under control, as this condition makes me fearful of falling ill again. I pay close attention to my health and usually undergo a regular physical examination every year*. *(P8)*

*I will pay attention to my diet by ensuring it provides the necessary nutrition for my body. I also plan to learn about traditional Chinese medicine to better support my recovery. In particular, I will avoid foods with strong or heavy flavors that may hinder the recovery*. *(P11)*

#### 3.1.4 Self‐Rehabilitation and Exercise

Although ECMO survivors have met the criteria for discharge, full recovery will take time. Self‐rehabilitation and exercise play a crucial role in the recovery process. Participants were actively engaging in self‐management to support their long‐term health. Some participants said that rehabilitation treatment had promoted their physical recovery. One of them expressed their desire to try traditional Chinese massage to strengthen their leg muscles.

*I feel like I'm getting better, so I ask my wife to hold me up and take me for a slow walk. Afterward, I notice that I recover more quickly*. *(P4)*

*I am going to have a TCM massage treatment at the end of this month, hoping that combining the TCM treatment will help promote the recovery of my leg strength*. *(P9)*

*After leaving the hospital, my leg function was still poor, so I stayed at a rehabilitation hospital for a month. Now I can walk around my home and go downstairs, which makes me feel much better*. *(P13)*

#### 3.1.5 Family Economic Burden

In China, the high cost of ECMO treatment imposes a substantial economic burden on most patients, who often incur significant debt from medical expenses. This strain is compounded by protracted recovery periods that prevent many from working, leading to a sharp decline in household income.

*I was in the ICU for 28 days, which cost a lot of money*, *a huge expense for our rural family. Now our family's economic situation is depressed. My son couldn't support the household on his own, so I started looking for work to help ease the burden*. *(P4)*

*Now the primary task of the family might mainly be about paying off the debt. The ECMO treatment cost hundreds of thousands of yuan, and I have no idea when the debt can be repaid. I'm worried*. *(P13)*

### 3.2 Emotional Distress and Support

#### 3.2.1 Emotional Difficulties and Psychological Challenges

Participants' emotions are influenced by various factors, including challenges in physical rehabilitation, uncertainty about illness outcomes, physical impairments and difficulties in identity adjustment. These factors contribute to persistent negative emotions and expose survivors to multiple sources of psychological pressure.

*I was unwell before, and as a result, my grades declined. Now I'm re‐studying with the goal of entering a good university. However, I'm uncertain whether I can perform well in the college entrance examination or if my health will hold up throughout the process. This uncertainty leaves me feeling confused*. *(P3)*

*I no longer dare to exercise, fearing it might affect my health and lead to another hospital visit. I want to return to work, but right now I'm stuck at home. Being away from work for so long has made me feel disconnected from society and increasingly worthless*. *(P7)*

*After being discharged from hospital, I began to learn about ECMO. The more I learned, the more frightened I felt. My body is covered with scars and wounds, and I'm uncertain whether I'll fully recover. I'm afraid of scarring*. *(P11)*

*I began to fear what others might say. I'm effectively disabled, and people stare and make comments when they see me. In my current predicament, getting married has become a problem*. *(P13)*

*There will be mood fluctuations and low periods. Most of the time, life feels uninteresting because I'm unable to feel happiness, and perhaps sometimes I am numb*. *(P15)*

#### 3.2.2 Emotion Management

Although survivors experience significant psychological distress, some are able to actively engage in self‐regulation, effectively manage their emotions and confront life's challenges with a positive mindset.

*I've always enjoyed fishing. Since falling ill, I still go fishing once a week. It would make me feel better*. *(P2)*

*I often remind myself to stay in a positive mood, as maintaining a good mood helps ensure my health and reduces some of the pressure on my family*. *(P4)*

*Engaging in physical activities refreshes me and helps keep my mood positive. Even though I don't have a job and spend most of my time at home with a little pressure, I still maintain a good state of mind*. *(P8)*

#### 3.2.3 Support Perception

Survivors are profoundly impacted by the ECMO treatment. Whether during hospitalisation or in the rehabilitation process after discharge, however, they have received substantial support from their families, medical staff and local organisations, which has helped them feel warmer and more confident in facing the challenges of recovery.

*My children have been very kind, telling me not to overthink. At home, my wife looks after me with great care. Thanks to my family's support, I feel more at ease and no longer see myself as a burden. I'm determined to recover soon*. *(P9)*

*The doctors and nurses saved my life and told me to come back on time after discharge, since I had traveled from another province for treatment. They gave me a detailed list of precautions. When I asked questions in the WeChat group, they always answered with such patience*. *I'm truly grateful to them*. *(P12)*

*When I was treated in the hospital, I didn't know which unit they belonged to, but they sent the money to my home and thanks to this money they saved my life*. *(P6)*

*The village has established a poor household assistance program for us, providing certain economic subsidies. I am very grateful to the government*. *(P13)*

### 3.3 Expectations and Needs

#### 3.3.1 Desire for Expanded Insurance Reimbursement

ECMO survivors face economic hardship and advocate for healthcare policies that favour economically disadvantaged groups. However, medical insurance reimbursement amounts are limited; the reimbursement rate of the New Rural Cooperative Medical Insurance is limited and relatively low, and many consumables and components required for ECMO therapy are not covered, which aggravates their economic burden. Therefore, survivors call on the government to expand insurance coverage for ECMO and increase related subsidies.

*From what I understand, the medical insurance reimbursement is limited. It would be helpful if there could be an additional subsidy on top of the reimbursement rate*. *(P1)*

*I hope there will be no disease in the world, and I also hope that the medical policy can better support ordinary people like us who face economic difficulties*. *(P3)*

#### 3.3.2 Eagerness for Rehabilitation Knowledge

Most participants expressed a strong desire for support such as rehabilitation programs, illness guidance and counselling, which they deemed crucial for an effective recovery of both body and mind.

*In fact, I would particularly like to know what precautions I should take after being discharged from the hospital. I also strongly hope that the hospital could provide more educational materials about disease management. While I can search for information online, the content available on the Internet often lacks authority and reliability*. *(P3)*

*In addition, receiving rehabilitation nursing knowledge and disease counseling can enhance my overall healthcare experience and make me feel more comfortable*. *(P11)*

#### 3.3.3 Hope for Simplified Follow‐Up Procedures

Some participants mentioned that they had travelled from another city and expressed hope that the follow‐up procedures would be straightforward and clearly explained to minimise waiting times.

*The review process can sometimes take an entire day, imposing a considerable burden on patients, particularly those who have traveled from other provinces. It should not be overly complicated*. *(P11)*

#### 3.3.4 Normalisation of Regular Follow‐Up

Participants demonstrated a positive attitude toward participating in follow‐up communication and hoped for its consistent implementation.

*Hearing that you are from Tongji conducting a telephone follow‐up, I feel reassured and happy. The call has truly lifted my mood*. *(P2)*

*It has been many years since I went on ECMO, and I'm so happy you're here for the follow‐up! Please keep in contact with me usually. I can ask you any questions and that will really help with my recovery*. *(P6)*

## 4 Discussion

This phenomenological study shows the lived experiences and feelings of ECMO survivors after ECMO treatment. Our findings reveal that ECMO survivors faced multifaceted challenges after discharge, including physical impairments, psychological distress and social reintegration difficulties.

The physical recovery process presents significant hurdles for most survivors. Our study indicates that initial mobility is severely compromised, with many patients unable to walk independently and some requiring wheelchair assistance. These limitations likely stem from prolonged immobilisation and intubation‐related injuries, resulting in muscle weakness and delayed functional recovery. While early rehabilitation has demonstrated potential benefits for physical function, muscle strength, mobility and independence, the current evidence remains limited by methodological constraints [26, 27]. This underscores the need for more robust, standardised approaches to physical rehabilitation.

The psychological impact of ECMO survival emerges as equally profound. Our participants reported frequently facing persistent emotional challenges, which is in line with the findings of Guo et al. [12], Minion et al. [19], and Chommeloux et al. [28] Multiple factors contribute to this psychological distress, including arduous physical recovery, substantial economic burdens from medical expenses and feelings of self‐blame and denial. These findings emphasise that psychological well‐being is not merely a secondary concern but fundamentally influences both early recovery and long‐term life satisfaction. Early identification and management of mental health issues are therefore crucial for improving their HRQoL [9], necessitating active family engagement in providing targeted emotional support across different recovery stages.

Social reintegration represents another critical dimension of recovery. While some survivors reported that reintegration into society positively influenced their psychological recovery, which corroborates with the findings of Hsieh et al. [29], social participation was identified as a main factor influencing HRQoL. Difficulties in resuming previous daily routines led to reduced participation in work, leisure activities and household responsibilities. Existing literature confirms these challenges, indicating that 25% of ECMO patients cannot return to work, while 44% experienced negative impacts on their careers [29, 30]. These findings suggest that healthcare providers should collaborate with community resources to offer vocational assessments and psychological support, thereby promoting resilience and facilitating social integration.

In this study, participants emphasised the importance of rehabilitation knowledge and guidance and that medical staff should provide patients with diversified rehabilitation plans, which is consistent with the findings of Sava et al. [16] Currently, the rehabilitation plan is still in its infancy, lacking standardised implementation frameworks [16]. The development of effective rehabilitation plans depends on multidisciplinary teamwork, an approach emphasised by the second Symposium of American Cancer Society as essential for comprehensive assessment, management and rehabilitation plan [27]. Available evidence suggests that early rehabilitation is safe and feasible primarily in specialised ECMO centres with experienced senior physical therapists and multidisciplinary collaboration [27]. It is recommended to develop standardised, evidence‐based rehabilitation guidelines that offer authoritative recovery guidance for survivors. Strengthening multidisciplinary collaboration is essential to creating individualised rehabilitation plans tailored to patients' specific needs.

Survivors, especially those coming from another province, expressed significant concerns about the current follow‐up examination. Many found the revisit process overly complicated. As a result, they voiced a strong preference for simplified procedures and more consistent communication with healthcare providers. These findings highlight the necessity of implementing multimodal strategies that integrate digital health solutions to overcome geographical barriers and enhance access to specialist support. To enhance continuity of care, which is essential for recovery, local healthcare systems should establish structured routine follow‐up mechanisms. Additionally, they should promote the use of self‐help rehabilitation manuals and support survivors in identifying and managing adverse psychological symptoms [31]. By ensuring sustained, evidence‐based services, healthcare providers can play a pivotal role in advancing comprehensive physical and psychological recovery.

## 5 Implications for Practice

The lived experience of ECMO patients is complex. Rehabilitation is protracted, requiring comprehensive support for societal reintegration. Understanding these experiences can guide nursing practice and alleviate patients' suffering.

## 6 Limitations

This study was conducted within specific regional contexts, and regional variations (e.g., variations in healthcare systems, resource availability, cultural factors or patient demographics) may introduce confounding and limit the external validity of our results. Furthermore, the exclusion of individuals with cognitive or psychiatric disorders, while methodologically necessary for our phenomenological approach, means that our findings do not represent the experiences of survivors who developed post‐ICU psychological sequelae such as PTSD. Future research should specifically investigate the lived experiences of this important patient subgroup.

## 7 Conclusion

Through qualitative interviews, this study explores the survival experiences of ECMO survivors, identifying three core themes: living conditions and challenges, emotional distress and support and expectations and needs. After discharge, ECMO survivors encounter significant difficulties in physical rehabilitation, psychological adjustment and access to adequate social support. Their overall quality of survival remains low, highlighting an urgent need for comprehensive external support and targeted interventions.

A multidisciplinary team should thoroughly understand the needs of ECMO survivors and develop scientific, feasible rehabilitation plans. Regular follow‐ups should be conducted to ensure continuity of care. Concurrently, timely evaluation of survivors' psychological status is essential, with continuous attention to their mental health. Furthermore, establishing a multidimensional support platform for patients and their families is recommended to deliver comprehensive social support, thereby enhancing the survival experience and HRQoL of ECMO survivors.

## Author Contributions

Meng Zhang: Conceptualisation; methodology; investigation; formal analysis; writing – original draft. Anqi Sun: Conceptualisation; methodology; data curation; formal analysis; writing – original draft. Anqi Yu: Methodology; investigation; formal analysis; writing – original draft. Juan Deng: Formal analysis; writing – review; editing. Yi Wang and Xiao Cheng: Supervision; formal analysis. Jie Xiong: Supervision; writing – review and editing.

## Funding

The authors have nothing to report.

## Ethics Statement

This study has been approved by the Ethics Committee of Tongji Hospital, Tongji Medical College, Huazhong University of Science and Technology (Ethics number: TJ‐IRB20230730) (Approval Date: 4 July 2023).

## Consent

Participants volunteer to participate and have the right to withdraw at any time. Prior to the interview, all respondents signed informed consent forms to participate in the interview and provide demographic data.

## Conflicts of Interest

The authors declare no conflicts of interest.

## Supporting information


**Appendix S1:** Interview Guide.

## Data Availability

Research data are not shared.
