# Supplementary material for: Lived Experience of Extracorporeal Membrane Oxygenation Survivors: A Phenomenological Study
Source: Nurs Crit Care. 2026 Feb 1;31(2):e70341. doi: 10.1111/nicc.70341 (PMC12862116; doi:10.1111/nicc.70341)
Supplement: Supplementary file 1 — Appendix S1: Interview Guide. [file NICC-31-0-s001.docx]

**Appendix**

**Supplemental File**

**Interview Guide**

| **Number** | **Questions** |
| --- | --- |
| 1 | Since ECMO treatment, what is your current physical condition and what are the differences between you and before? |
| 2 | How does the change of your physical condition affect your normal life? |
| 3 | How do you feel about your emotions? What situations affect your emotions? |
| 4 | Have you ever felt pressure or encountered difficulties? What situations cause you stress or difficulty? |
| 5 | How did you deal with the existing difficulties and pressures? |
| 6 | How is your work (study) and what are the causes? |
| 7 | How did your family, friends (classmates) and medical staff help you? What do you want your health care provider to help you with? |
| 8 | What is your spiritual support or religious belief? In what ways has it helped you? |
| 9 | What do you think can help you improve your experience after ECMO treatment? |
| 10 | Is there anything else you would like to add or share about your experience ? |
